# Health Equity in the Veterans Health Administration From Veterans’ Perspectives by Race and Sex

**DOI:** 10.1001/jamanetworkopen.2023.56600

**Published:** 2024-02-19

**Authors:** Natalie S. Lee, Shimrit Keddem, Anneliese E. Sorrentino, Kevin Ahmaad Jenkins, Judith A. Long

**Affiliations:** 1 Division of General Internal Medicine, The Ohio State University Wexner Medical Center, Columbus; 2 Center for the Advancement of Team Science, Analytics, and Systems Thinking in Health Services and Implementation Science Research, The Ohio State University, Columbus; 3 Center for Health Equity Research and Promotion, Corporal Michael J. Crescenz Veterans Affairs Medical Center, Philadelphia, Pennsylvania; 4 Department of Family Medicine and Community Health, University of Pennsylvania Perelman School of Medicine, Philadelphia; 5 Leonard Davis Institute of Health Economics, University of Pennsylvania, Philadelphia; 6 Division of General Internal Medicine, Department of Medicine, University of Pennsylvania Perelman School of Medicine, Philadelphia

## Abstract

**Importance:**

Advancing equitable patient-centered care in the Veterans Health Administration (VHA) requires understanding the differential experiences of unique patient groups.

**Objective:**

To inform a comprehensive strategy for improving VHA health equity through the comparative qualitative analysis of care experiences at the VHA among veterans of Black and White race and male and female sex.

**Design, Setting, and Participants:**

This qualitative study used a technique termed *freelisting*, an anthropologic technique eliciting responses in list form, at an urban academic VHA medical center from August 2, 2021, to February 9, 2022. Participants included veterans with chronic hypertension. The length of individual lists, item order in those lists, and item frequency across lists were used to calculate a salience score for each item, allowing comparison of salient words and topics within and across different groups. Participants were asked about current perceptions of VHA care, challenges in the past year, virtual care, suggestions for change, and experiences of racism. Data were analyzed from February 10 through September 30, 2022.

**Main Outcomes and Measures:**

The Smith salience index, which measures the frequency and rank of each word or phrase, was calculated for each group.

**Results:**

Responses from 49 veterans (12 Black men, 12 Black women, 12 White men, and 13 White women) were compared by race (24 Black and 25 White) and sex (24 men and 25 women). The mean (SD) age was 64.5 (9.2) years. Some positive items were salient across race and sex, including “good medical care” and telehealth as a “comfortable/great option,” as were some negative items, including “long waits/delays in getting care,” “transportation/traffic challenges,” and “anxiety/stress/fear.” Reporting “no impact” of racism on experiences of VHA health care was salient across race and sex; however, reports of race-related unprofessional treatment and active avoidance of race-related conflict differed by race (present among Black and not White participants). Experiences of interpersonal interactions also diverged. “Impersonal/cursory” telehealth experiences and the need for “more personal/attentive” care were salient among women and Black participants, but not men or White participants, who associated VHA care with courtesy and respect.

**Conclusions and Relevance:**

In this qualitative freelist study of veteran experiences, divergent experiences of interpersonal care by race and sex provided insights for improving equitable, patient-centered VHA care. Future research and interventions could focus on identifying differences across broader categories both within and beyond race and sex and bolstering efforts to improve respect and personalized care to diverse veteran populations.

## Key Points

**Question:**

How do perceptions of veterans’ health care and recommendations for change differ by race and sex?

**Findings:**

This qualitative study of veterans of Black or White race and male or female sex used a semiquantitative technique termed *freelisting* to compare salient themes in experiences of care after 2020 among 49 US veterans with chronic hypertension. While positive perceptions of care quality were common, perceived quality of experiences and a need to improve them differed by race and sex.

**Meaning:**

Focusing on the quality of interpersonal interactions that occur within the Veterans Health Administration may represent an important opportunity for addressing inequity, especially for Black and women veterans.

## Teaser

This qualitative study assesses veterans’ perceptions and experiences of Veterans Health Administration (VHA) care by race and sex to understand what steps need be taken to improve equitable patient-centered care.

## Introduction

Equitable care is a major tenet of the Veterans Health Administration (VHA) mission.^1^ However, disparities persist in health and health care due to nonclinical differences such as race and sex.^2,3,4,5,6^ Understanding veteran experiences is an essential part of the VHA action plan for advancing health equity.^7^

Veteran experiences must be contextualized within the realities of the contemporary sociopolitical environment. The past several years were characterized by multiple layers of tumult. The COVID-19 pandemic interacted in complex ways with racism, health, and health care, exposing and exacerbating disparities in health care and health outcomes.^8,9^ Telehealth suddenly became a dominant health care delivery medium,^10^ which improved access for some but further excluded those with less access to digital health tools.^11^ There was heightened media visibility of racial and ethnic, social, and political tensions and stresses, including within health care, which can affect veterans’ perceptions of care, trust in their care, and subsequent use of health care services.^12,13^ Not only do these contexts inform individual experiences of care, but those already in marginalized positions are likely also to experience differential burdens of external stressors.^14^ Thus, as the pandemic accelerated rupturing of existing fissures in health equity, we anticipated new or further rifts in experiences between individuals within and outside mainstream groups.

Therefore, the purpose of this study was to conduct an up-to-date assessment of veteran perceptions and experiences of VHA care by race and sex in the context of recent events, toward the broader goal of understanding what steps need be taken to improve equitable patient-centered care in the current environment. Patient satisfaction measures are one way to assess and analyze health care quality and experience,^15,16,17^ and one such measure in the VHA is the Survey of Health Care Experiences of Patients quantitative survey.^18,19^ However, survey-based approaches can limit the depth of our understanding of the patient experience^20^ and can also limit the breadth of response options to predefined concepts. Therefore, we used *freelisting*,^21^ a qualitative technique used in anthropology, as a simple and reproducible approach to capture veteran experiences and priorities, including potentially unimagined or uncategorized concepts, in their own words.^21^ This comparative approach also provides nuanced insights into veteran experiences based on what is said vs unsaid in different groups. We focused on veterans with hypertension because of known disparities in the prevalence and treatment of high blood pressure,^22,23^ and because it is a chronic condition that requires regular VHA interactions. While disparities across many racial, ethnic, sex, and sociodemographic lines must all be addressed, we elected to examine Black and White and male and female binary categories as a critical starting point.

## Methods

This study was reviewed and approved by the institutional review board of the Corporal Michael J. Crescenz Veterans Affairs Medical Center. All participants provided oral informed consent. This study followed the Standards for Reporting Qualitative Research (SRQR) reporting guideline.

### Study Sample

In freelisting, a minimum sample of 20 participants is required in each analytic group to discern salience.^24^ To enable analyses by sex and race, we aimed to recruit 48 veterans receiving primary care at the Corporal Michael J. Crescenz Veterans Affairs Medical Center clinics in Philadelphia, Pennsylvania, with 12 participants in each group of Black men, Black women, White men, and White women; we overenrolled by 1 participant (a White woman) due to administrative error. We limited comparison to White and Black race only because Black veterans are the largest racial minority group in the VHA,^1^ and the Black-White dynamic was among those highly publicized during the COVID-19 pandemic. All participants had an active diagnosis of hypertension based on *International Statistical Classification of Diseases and Related Health Problems, Tenth Revision*, codes. We focused on patients with actively managed hypertension at the VHA prior to the first wave of the COVID-19 pandemic; to that end, all participants were prescribed and had been taking at least 1 antihypertensive through the local VHA since December 1, 2019, or earlier and had established outpatient care at the local VHA prior to June 1, 2018. All participants were able to read and write English and make their own health care decisions. We excluded patients receiving cancer treatment or hospice care, who were pregnant, or who had advanced kidney disease (glomerular filtration rate of ≤45 mL/min/1.73 m^2^) because these conditions change how hypertension is managed.

Using the Veterans Affairs Informatics and Computing Infrastructure and the Corporate Data Warehouse, we identified a cohort of potentially eligible participants based on race, sex, diagnosis of hypertension, and use of antihypertensive therapy and who had established care at the VHA prior to June 1, 2018, as documented in the electronic health record (EHR). Race in the VHA EHR is self-identified information collected during enrollment with the VHA or at an outpatient or inpatient VHA encounter^25^; the options are American Indian or Alaska Native, Asian, Black, Native Hawaiian or Other Pacific Islander, and White. We did not use ethnicity data because this was beyond our stated scope. Sex as identified in the VHA EHR was based on sex assigned at birth. Participants verified demographic information, including race, sex, and gender identity during telephone screening interviews.

The research team mailed recruitment packets consisting of recruitment letters and oral informed consent form scripts to random groups of 100 patients at a time; recruitment letters were divided equally across Black men and women and White men and women (25 letters each). The letter directed interested potential participants to call the research telephone line. After 1 week, the team also called potential participants who had not responded to the mailed letters. If there was no response, we made no more than 1 additional telephone call. After a minimum of 3 weeks, we mailed out the next batch of 100 letters. We further assessed eligibility through telephone screening and obtained verbal consent for participation.

### Interview Guide Development

The entire research team (1 Asian woman [N.S.L.], 3 White women [S.K., A.E.S., and J.A.L.], and 1 Black man [K.A.J.]) provided input in developing the interview guide, which consisted of 6 freelisting questions and 12 additional questions relating to military service, use of health care services, and other sociodemographic information, including a validated VHA screening question to assess risk for homelessness,^26^ as well as questions drawn from the 2021 National Health Interview Survey Questionnaire from the Centers for Disease Control and Prevention National Center for Health Statistics to assess income and public assistance.^27^ An investigator (N.S.L.) piloted freelist questions in person with a convenience sample of patients at the local VHA medical center.

### Freelisting

Freelisting is a qualitative approach with roots in anthropology that shares features of both quantitative surveys and more traditional qualitative interviews. Participants are first asked a question, then asked to list all words and comments that first come to mind in response. The goal is to elicit spontaneous responses to interview questions, allowing researchers to understand how specific populations define health-related concepts.^24^

Freelisting is an increasingly popular tool for health services research; applications range from understanding local asthma triggers to identifying patient and family priorities in the intensive care unit.^28,29^ As a qualitative technique, the final analytic product preserves concepts in the patient’s own words. Through calculation of a salience index (further described in the statistical analysis section), freelisting accounts for the ubiquity of an item (how often it occurs) across individuals in a group, as well as the item’s salience (cognitive accessibility or importance) to the group, which allows for comparison of responses across different groups. For example, freelisting analyses have been used to identify racial differences in perceptions of treatment in Alzheimer disease and in psoriasis.^30,31^ Additionally, freelisting is quick, reducing potential time burden on participants and permitting larger sample sizes.

### Data Collection

The principal investigator (N.S.L.) and research coordinator (A.E.S.) completed all interviews via telephone between August 2, 2021, and February 9, 2022. Six interviews were completed in 2022. Participants consented to have their interviews audiorecorded and transcribed.

Interviewers introduced participants to the freelisting exercise with an unrelated practice question, then proceeded to the 6 research freelisting questions. Participants had as much time as desired for each question and were asked to clarify unclear responses only after completing their lists. The described interview process typically took 25 minutes.

### Statistical Analysis

Data were analyzed from February 10 through September 30, 2022. Three investigators carefully cleaned all freelist data for analysis according to standard procedures.^24^ Two investigators (a primary care physician and health services researcher [N.S.L.] and a licensed marriage and family therapist and research coordinator with extensive qualitative research experience [A.E.S.]) collaboratively cleaned the raw lists to combine root words (eg, “frustrated” and “frustrating”), synonyms (eg, “happy” and “glad”), and similar concepts (eg, “excellent doctors” and “professional in their craft”) into grouped terms, staying as close to participants’ words as possible. As a balancing measure commonly used in freelisting,^24,32^ a third investigator (a health services researcher with expertise in freelisting and other qualitative methods [S.K.]) reviewed the grouped terms during interim analysis, and all authors reviewed and contributed to the revision of the final list of grouped terms.

We analyzed the final cleaned freelists using the AnthroTools package in R Studio, version 2022.02.3 Build 492 (R Project for Statistical Computing). At the individual level, each item (grouped term) on a given list is assigned a salience score, which is calculated by taking the order in which an item is listed, inversely coding the order number (eg, an item listed 1 of 5 is coded 5), and dividing that number by the total number of items on the individual’s list; thus, the first item on an individual’s list receives a salience score of 1. Individual lists are then combined into a group list, and each item is given a Smith salience index, calculated by dividing the sum of salience scores for the item by the total number of participants. The standard equation for Smith salience index is ( ∑ s)/N, where s is individual item salience and N is the group sample size.^21^ The Smith salience index therefore captures word frequency and rank in a population of interest; the index ranges between 0 and 1, with words mentioned often and early having higher indices. Three team members (N.S.L., A.E.S., and S.K.) reviewed the overall freelist results for each question (ie, the distribution of words and their measures of salience) with an eye toward identifying grouped terms that were too narrow or broad. Once we finalized the overall item lists, we proceeded with the analysis by groups (ie, race and sex).

## Results

Three hundred veterans were contacted by mail (Figure). Of the 69 potential participants screened, 49 were enrolled. The remaining 20 participants declined (n = 3), were ineligible (n = 5), or were unable to be contacted subsequently (n = 12). Forty-eight participants completed all interview components. One White female participant did not complete freelist questions 5 and 6 due to emotional distress, but she completed all other interview components; she was excluded from the analysis for those 2 questions.

**Figure.**
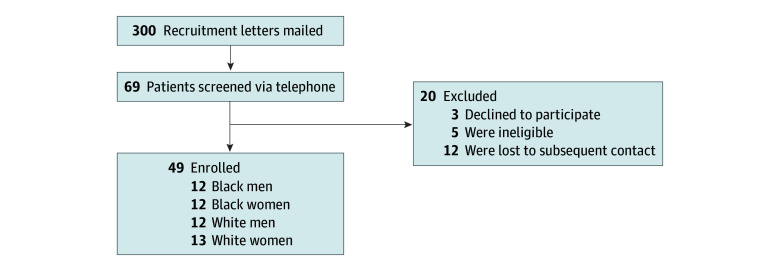
Study Flow Diagram

The mean (SD) age of participants was 64.5 (9.2) years, and 28 (57.1%) had served in the US Army; some reported having served in multiple branches. A minority of participants had contracted COVID-19 (5 [10.2%]) or were hospitalized (8 [16.3%]) between 2020 and the time of interview. Most had stable housing (48 [98.0%]). Four participants reported receiving public assistance in the past year; 3 of these reported receiving assistance for food (Table 1).

**Table 1.** Characteristics of Participants Who Completed Freelist Interviews

Characteristic	Participant group^a^				
	All (N = 49)	Black men (n = 12)	Black women (n = 12)	White men (n = 12)	White women (n = 13)
Age, mean (SD), y	64.5 (9.2)	64.0 (7.5)	60.5 (9.8)	71.3 (9.5)	60.5 (6.4)
Military branch^b^					
Army	28/49 (57.1)	8/12 (66.7)	8/12 (66.7)	4/12 (33.3)	8/13 (61.5)
Navy	14/49 (28.6)	3/12 (25.0)	3/12 (25.0)	6/12 (50.0)	2/13 (15.4)
Coast Guard	1/49 (2.0)	1/12 (8.3)	0	0	0
Marines	2/49 (4.1)	0	0	1/12 (8.3)	1/13 (7.7)
Air Force	6/49 (12.2)	1/12 (8.3)	1/12 (8.3)	1/12 (8.3)	3/13 (23.1)
Range of dates of service	1952-2012	1965-2006	1965-2012	1952-2012	1965-2007
Contact with health care during the study period					
Contracted COVID-19	5/49 (10.2)	3/12 (25.0)	0	2/12 (16.7)	0
Hospitalized (VHA or non-VHA)	8/49 (16.3)	3/12 (25.0)	2/12 (16.7)	3/12 (25.0)	0
Total hospitalizations, mean (SD)	1.3 (0.5)	1.3 (0.6)	1.0 (0)	1.3 (0.6)	0
Used ED services (VHA or non-VHA), %	22/49 (44.9)	9/12 (75.0)	6/12 (50.0)	3/12 (25.0)	4/13 (30.8)
Total ED visits, mean (SD)	2.0 (1.2)	2.3 (1.1)	1.7 (0.5)	1.3 (0.6)	2.5 (2.4)
Changed health care clinician	9/49 (18.4)	1/12 (8.3)	1/12 (8.3)	2/12 (16.7)	5/12 (41.7)
Changed clinician away from VHA	1/9 (11.1)	0	0	0	20.0 (1 of 5)
Living in stable housing	48/49 (98.0)	11/12 (91.7)	12/12 (100)	12/12 (100)	13/13 (100)
Worried or concerned about stable housing	2/49 (4.1)	1/12 (8.3)	1/12 (8.3)	0	0
Income before taxes, mean (SD), US$	70 062 (47 199)	63 575 (62 822)	83 375 (35 128)	71 236 (44 987)	60 851 (46 050)
Declined to answer	4/49 (8.2)	1/12 (8.3)	0	1/12 (8.3)	2/13 (15.4)
Received public assistance	4/49 (8.2)	1/12 (8.3)	1/12 (8.3)	0	2/13 (15.4)

Abbreviations: ED, emergency department; VHA, Veterans Health Administration.

^a^ Unless otherwise indicated, data are expressed as No./total No. (%) of participants.

^b^ Some participants served in multiple military branches.

Freelist results are presented by question. The top 5 most salient answers are presented in Table 2, which is a common method of presenting freelist data^24^; only 3 responses are listed for question 6 because few concepts were generated by respondents.

**Table 2.** Responses to Freelist Interview Questions by Sex and Race

Sex				Race			
Women		Men		Black veterans		White veterans	
Response	SSI^a^	Response	SSI^a^	Response	SSI^a^	Response	SSI^a^
**1. Please make a list of all the feelings that come to mind right now when you think about receiving health care at the VHA.**							
Not timely/long wait	0.29	Like	0.38	Good medical care	0.27	Like	0.40
Anxiety/stress/fear	0.26	Convenient/efficient/helpful	0.26	Convenient/efficient/helpful	0.27	Anxiety/stress/fear	0.32
Like	0.25	Good medical care	0.25	Like	0.23	Nice service/courteous/respect	0.25
Good medical care	0.23	Nice service/courteous/respect	0.23	Not timely/long wait	0.20	Good medical care	0.21
Convenient/efficient/helpful	0.20	Anxiety/stress/fear	0.20	Anxiety/stress/fear	0.14	Convenient/efficient/helpful	0.19
**2. The past year has been challenging for many people, for many different reasons—the pandemic, increased social unrest, issues of racism, loss of income, unemployment, just to name a few. With that in mind, please list all the challenges you faced in receiving health care at the VHA in the past year.**							
No/limited in-person care	0.33	Few/no challenges	0.30	Long waits/delays in getting care	0.36	No/limited in-person care	0.27
Long waits/delays in getting care	0.31	Long waits/delays in getting care	0.29	Few/no challenges	0.29	Long waits/delays in getting care	0.24
Few/no challenges	0.16	No/limited in-person care	0.17	No/limited in-person care	0.23	Few/no challenges	0.16
Feel unheard/ignored/shut down/uncared for	0.12	Transportation/traffic challenges	0.11	Transportation/traffic challenges	0.10	Transportation/traffic challenges	0.11
Transportation/traffic challenges	0.10	Having to use teleconferences (telephone/video) instead of in-person consultations	0.10	Delay in getting medications by mail	0.09	[Hard] to get somebody on the phone	0.10
**3. Many VA services were and are being offered virtually, meaning over telephone or video on a computer or smartphone, rather than in person. List your feelings or attitudes toward virtual visits at the VHA.**							
Good experience/comfortable/great option	0.27	Good experience/comfortable/great option	0.37	Good experience/comfortable/great option	0.32	Good experience/comfortable/great option	0.31
Beneficial/helpful/supportive	0.21	Prefer in-person	0.19	Beneficial/helpful/supportive	0.23	Limit doctor decision making/subpar [care]	0.19
Impersonal/cursory/feel like a number	0.18	Went well/no problems	0.17	Prefer in-person	0.21	Dislike virtual/negative experience	0.17
Dislike virtual/negative experience	0.17	Technology problems on both sides	0.17	Technology problems on both sides	0.19	Convenient/easier/time-saving	0.17
Convenient/easier/time-saving	0.16	Convenient/easier/time-saving	0.13	Impersonal/cursory/feel like a number	0.14	Appropriate for some visits/have their place	0.16
**4. List all the changes you want to see in VHA health care after this last year.**							
In-person visits	0.20	Faster/more accessible appointments	0.27	Faster/more accessible appointments	0.15	Faster/more accessible appointments	0.21
Improve hospitality	0.15	No complaints	0.23	More personal care/attentive to emotion/finances/needs	0.12	No complaints	0.20
More personal care/attentive to emotion/finances/needs	0.12	Live people answer phones rather than recorded messages	0.10	Live people answer phones rather than recorded messages	0.11	In-person visits	0.20
More satellites/services	0.12	Hire more support staff and doctors	0.08	More satellites/services	0.10	Improve hospitality	0.15
Faster/more accessible appointments	0.10	More personal care/attentive to emotion/finances/needs	0.08	More one-on-one with doctor	0.10	Keep/add virtual options	0.13
**5. Please list all the ways the issues of race and racism in the past year impacted you.**							
No impact	0.29	No impact	0.68	No impact	0.43	No impact	0.54
Discriminated/treated differently	0.27	Discriminated/treated differently	0.16	Discriminated/treated differently	0.32	Anger/frustration at [lack of] change	0.11
Emotionally/mentally disturbing	0.21	Anger/frustration at [lack of] change	0.11	Anger/frustration at [lack of] change	0.16	Discriminated/treated differently	0.10
Anger/frustration at [lack of] change	0.16	Keep low profile/stay out of it	0.06	Emotionally/mentally disturbing	0.15	Emotionally/mentally disturbing	0.10
Keep low profile/stay out of it	0.05	More awareness/understanding	0.05	Keep low profile/stay out of it	0.09	More awareness/understanding	0.08
**6. Please list all the ways the issues of race and racism in the past year impact your experience of health care at the VHA.**							
No impact/not experienced or observed	0.75	No impact/not experienced or observed	0.95	No impact/not experienced or observed	0.83	No impact/not experienced or observed	0.86
Treated differently because of my race	0.17	Unprofessional treatment	0.04	Treated differently because of my race	0.09	Treated differently because of my race	0.10
Unprofessional treatment	0.04	Treated differently because of my race	0.02	Unprofessional treatment	0.08	Unprofessional treatment	0.0

Abbreviations: SSI, Smith salience index; VHA, Veterans Health Administration.

^a^ Calculated by dividing the sum of salience scores for the item by the total number of participants. The index ranges between 0 and 1, with words mentioned often and early having higher indices.

### Question 1: List of All the Feelings That Come to Mind Right Now When You Think About Receiving Health Care at the VHA

Across all groups, salient positive themes were “like,” “good medical care,” and “convenient/efficient/helpful;” a negative theme of “anxiety/stress/fear” had shared salience. Among women and Black respondents, long waits had a salient association with VHA care. Courtesy and respect were salient for White but not Black respondents and men but not women.

### Question 2: List All the Challenges You Faced in Receiving Health Care at the VHA in the Past Year

Common salient challenges were limitations to in-person care, delays in care, and challenges related to traffic or transportation. There was also a salient sense that there were few to no challenges in receiving care. Other challenges were salient by race and sex. Women felt unheard; using teleconference instead of in-person care was challenging for men; difficulty connecting with a live person on the telephone was salient for White veterans; and delays in receiving medication by mail was salient for Black veterans.

### Question 3: List Your Feelings or Attitudes Toward Virtual Visits at the VHA

“Good experience/comfortable/great option” was a salient concept for all groups. Other positive themes were salient by groups; White men and women, but not Black veterans, identified convenience. Men identified smooth logistics (“went well”), and for Black veterans and women, there was a sense of added value (“beneficial”). Technology problems were salient by race (Black) and sex (men). For women and for Black veterans—but not White veterans or men—a salient concept was that virtual care felt impersonal or cursory.

### Question 4: List All the Changes You Want to See in VHA Health Care After This Last Year

The desire for faster and more accessible appointments was salient for all groups. For women and Black participants, high-salience concepts included the desire for more personalized care and expanded satellites and services. For women and White veterans, but not Black veterans or men, an identified need for improved hospitality was salient. Meanwhile, “no complaints” was salient for veterans who were men and White.

### Question 5: List All the Ways the Issues of Race and Racism in the Past Year Impacted You

The most salient answer for all was no impact. All groups also reported experiences of discrimination. The idea of keeping a “low profile” and avoiding conflict was not salient for White veterans but salient for other groups, suggesting this was most salient for Black men and women.

### Question 6: List All the Ways the Issues of Race and Racism in the Past Year Impacted Your Experience of Health Care at the VHA

The most salient response was no impact; the high salience index of this response suggests this was the first-order answer for many respondents, and the overall brevity of the lists suggests there was little variation in this experience across groups. “Unprofessional treatment” was a low salience concept for most groups; however, it was absent among White participants.

## Discussion

In this qualitative study, we used freelisting to explore veterans’ perspectives of VHA care by race and sex, especially following the marked tumult that coincided with the COVID-19 pandemic. Our results are consistent with those of other literature on perceived strengths and areas for improvement in the VHA, as discussed below. Our comparative qualitative approach allows subtle but important differences in salient themes to emerge by race and sex and thereby inform ongoing efforts to better serve veterans.

Overall, there were shared positive elements of VHA care, consistent with prior findings that suggest generally high levels of satisfaction with VHA health care across race and ethnicity and sex.^3^ “Like” was a salient sentiment across all groups, with many identifying few to no challenges in receiving VHA care even during the turbulence of the pandemic years. Salient associations with the VHA such as “good medical care” and convenience suggest these are high-priority, well-appreciated features of VHA care. General satisfaction with telehealth as a care option among our participants is also consistent with the literature,^33^ though there were differences in the salience of perceived shortcomings.

Some of the negative aspects of care identified are also consistent with known areas for VHA improvement. Salient themes in this analysis such as long waits for care that were exacerbated by pandemic-related disruptions and the need to improve accessibility and satellite services, are well-documented problems within the VHA.^3,34^ Challenges related to facilities, such as traffic and transportation, have also been cited in other studies.^35^ Notably, associations of the VHA with anxiety, stress, and fear were salient for all groups. It is unclear whether these were responses to the extraordinary circumstances at the height of the pandemic or more deeply rooted experiences of care at the VHA. Given common salience in this study, additional efforts may be needed to understand and address drivers of anxiety and stress in receiving VHA care.

Salient themes in the experiences of interpersonal interactions diverged by race and sex. Women and Black veterans noted a sense of interpersonal distance in their VHA interactions. Specifically, women identified the need for improving hospitality and personal, whole-person care at the VHA as priorities for change, and related to that, their salient experiences included feeling unheard during the height of the pandemic and telehealth interactions that felt impersonal. Similar sentiments were salient among Black veterans, who also identified a need for more human interactions with clinicians and staff, rather than with automated recordings. In contrast, there were experiences unique to veterans who were White and men; these included perceived courtesy and respect at the VHA, few problems with the transition to telehealth, and contentment with the VHA status quo. Unprofessional treatment related to race was cited by men, women, and Black veterans but absent among White participants, suggesting that only men and women who also self-identified as Black perceived discrimination in this form.

These findings are consistent with other studies of patient experience by race and ethnicity and sex.^18,35,36^ In one study,^18^ Black and Hispanic veterans reported less frequent experiences considered positive across several domains (eg, getting needed care, getting care quickly, clinical communication), and more frequent experiences considered negative, relative to White non-Hispanic veterans. Another study that explicitly included an item on satisfaction with respect by VHA clinicians and staff^3^ found an interaction between race and sex, with White women experiencing less respect than White men. Veterans from racial and ethnic minority groups are also more likely to report experiences of discrimination.^35,36^ Taken together, our findings contribute to evidence indicating that, both before and after the pandemic, there are enduring differences in the quality of interactions within the VHA by race and sex, with White men most likely perceiving more positive experiences relative to other groups.

The VHA is well-positioned to start implementing initiatives aimed at improving the experience of care for Black and women veterans, considering the relationship-centered approaches for improving VHA health advanced by the VHA Whole Health Program.^37^ These efforts should be further informed by understanding and addressing the quality of perceived interactions within the VHA. Improving interactions may also improve perceptions of discrimination.^35^ Our results also emphasize the importance of better understanding how different groups might experience respect and personal care, and how that can be translated across the VHA for both in-person and remote interactions. Our analytic approach did not allow explicit discernment regarding the need for improved interactions with clinical vs nonclinical staff, but the literature suggests it is likely both.^35,38,39^

Additionally, our findings hint at disparate experiences of care that accord with other studies. For example, in our study and others, experiences of delayed or untimely care differ by race.^5^ While telehealth was seen as a good option, the perception of technology problems differed by race (reported by Black participants) and sex (reported by men), implying a digital divide.^40^ This is consistent with other studies that suggest telehealth services impact disparities in complex ways–diminishing disparities in aspects of care access, but contributing to disparities in the receipt and satisfaction of care.^41,42,43,44,45^

### Strengths and Limitations

This freelist analysis allowed us to capture and compare salient concepts for men, women, and Black and White veterans in their own words. That we recruited fairly equal numbers across race and sex categories is a marked strength. The VHA medical center in this study is an urban practice affiliated with a large university hospital as well as local community-based outpatient clinics; thus, our findings should resonate with many VHA medical centers. Nevertheless, the study was limited to a single VHA site, which limits generalizability; prior national studies of VHA experiences have found that racial and ethnic differences in experiences could be attributed to between-facility variations.^18^ Our sample size allowed comparisons across race and sex; however, the study was not designed to formally discern the intersectionality of these categories (eg, the unique experiences of Black women who are vulnerable to both racism and sexism). We also focused on limited sex and racial categories, and similar studies are warranted across additional populations. In several questions, veterans answered requests for emotions or attitudes with broader concepts. We accepted these answers without redirection because we valued their unhindered, instinctive responses to the question. A limitation of freelisting is that the cutoff for determining salience (eg, top 5 or top 3 concepts) is subjective; thus, larger sample sizes and different cutoff criteria could yield modest differences in findings considered “salient.” Finally, prior research suggests that Black veterans who experience racism in health care downplay or suppress their emotions relating to racist events,^39^ and a top-of-mind approach like freelisting may have been inadequate to explore experiences of racism in depth.

## Conclusion

Overall, our results suggest that in addition to known issues with access, improving the quality of interactions within the VHA should be a priority, especially for women and racial minority individuals. Additional understanding of the unique multilevel barriers and facilitators of respectful, personal care experiences for various minoritized populations and relating those experiences to clinical hypertension outcomes is an important next step.

## Notes

Supplement.
Data Sharing Statement
