# Supplementary material for: Health Equity in the Veterans Health Administration From Veterans’ Perspectives by Race and Sex
Source: JAMA Netw Open. 2024 Feb 19;7(2):e2356600. doi: 10.1001/jamanetworkopen.2023.56600 (PMC10877456; doi:10.1001/jamanetworkopen.2023.56600)
Supplement: Supplement. — Data Sharing Statement [file jamanetwopen-e2356600-s001.pdf]

Data Sharing Statement

Lee. Health Equity in the Veterans Health Administration From Veterans' Perspectives by Race and Sex. *JAMA Netw Open*. Published February 19, 2024.
doi:10.1001/jamanetworkopen.2023.56600

Data
Data available: No

Additional Information
Explanation for why data not available: deidentified analytic files will be made available upon request
